# Perceived Simulation Design Quality, Educational Effectiveness, Satisfaction, and Self-Confidence in a Resource-Conscious Burn Care Simulation: A Multicentre Observational Study

**DOI:** 10.3390/ebj7040052

**Published:** 2026-10-09

**Authors:** William Meschial, Muriel Fernanda de Lima, Luciene Andrade, Alice Nespollo, Isadora Cardias, Cristiane Siebeneichler, Sandra Marin, Julia Bitencourt, Denise Zocche

**Affiliations:** 1 Postgraduate Program in Nursing, Santa Catarina State University (UDESC), Chapeco 89815-630, SC, Brazil; isadoracardias11@gmail.com (I.C.); cristiane.sieb@gmail.com (C.S.); sandrapeju@hotmail.com (S.M.); denise.zocche@udesc.br (D.Z.); 2 Undergraduate Nursing Programme, Integrated Health Institute, Federal University of Mato Grosso do Sul (UFMS), Campo Grande 79070-900, MS, Brazil; muriel.lima@ufms.br; 3 Undergraduate Nursing Programme, Institute of Health Sciences, Federal University of Mato Grosso (UFMT), Cuiaba 78060-900, MT, Brazil; luciene.andrade@ufmt.br (L.A.); alice.nespollo@ufmt.br (A.N.); 4 Postgraduate Program in Nursing, Federal University of Fronteira Sul (UFFS), Chapeco 89815-899, SC, Brazil; julia.bitencourt@uffs.edu.br

**Keywords:** burns, simulation training, students nursing, patient simulation, education nursing, self efficacy

## Abstract

**Highlights:**

**What are the main findings?**

- Higher perceived simulation design quality was associated with greater perceived educational effectiveness, satisfaction, and self-confidence.
- The burn care scenario achieved content validity and was implemented across three public universities using simulated patients, moulage, and readily available resources.

**What are the implications of the main findings?**

- Favourable perceived outcomes were observed with a structured scenario that did not require advanced simulation technologies.
- The scenario offers a structured, adaptable approach to initial burn care training in settings with limited access to advanced simulation technologies.

**Abstract:**

**Background:** Early recognition and management of burns are essential, yet access to technologically complex simulation may be limited in resource-constrained educational settings. **Methods:** We developed a resource-conscious simulation scenario for the initial management of adult burns, established its content validity, and evaluated its implementation across three Brazilian public universities. Nine experts assessed content validity, and post-intervention evaluation included 48 nursing students. Perceived simulation design quality, perceived educational effectiveness, satisfaction, and self-confidence were measured. **Results:** The scenario demonstrated adequate content validity (S-CVI/Ave = 0.86). Median scores were 4.60/5.0 for design quality, 2.63/3.0 for perceived educational effectiveness, 4.80/5.0 for satisfaction, and 4.38/5.0 for self-confidence. Perceived design quality correlated positively with perceived effectiveness (ρ = 0.557; 95% CI 0.317–0.731), satisfaction (ρ = 0.711; 95% CI 0.529–0.831), and self-confidence (ρ = 0.612; 95% CI 0.390–0.767; all *p* < 0.001). After adjustment for age, region, and previous simulation experience, each one-point increase in design rating remained associated with a 0.43-point increase in perceived effectiveness (95% CI 0.17–0.69; *p* = 0.002). **Conclusions:** Higher perceived design quality was associated with more favourable self-reported educational outcomes. The scenario was implemented across three institutions, but its effects on knowledge and clinical performance require further evaluation.

## 1. Introduction

Burn injuries remain a major global public health problem, accounting for approximately 180,000 deaths annually, while more than 11 million people require healthcare for burns each year. This burden falls disproportionately on low- and middle-income countries, where most burn-related deaths occur [1,2]. In Brazil, a national analysis of burn-related mortality between 2010 and 2019 found that 76.2% of deaths occurred among males and that electrical burns were associated with a 59% higher risk of pre-hospital death than thermal burns [3]. Regional studies have also demonstrated variation in injury mechanisms, affected populations, severity, and outcomes [4,5]. These findings reinforce the need for healthcare professionals who are adequately prepared to recognise burns and manage potentially life-threatening complications at an early stage.

The initial management of a person with burn injuries requires systematic assessment of burn extent and depth, early recognition of inhalation injury, advanced airway management, ventilatory support, prevention of hypothermia, adequate analgesia, accurate estimation of the total body surface area (TBSA) burned, and prompt initiation of fluid resuscitation, followed by safe transfer to a specialist burn centre. However, recent evidence indicates persistent gaps in the knowledge and skills of health profession students and healthcare professionals regarding the initial management of burn injuries, including fundamental aspects of care [6,7,8,9]. These deficiencies may delay critical interventions and compromise patient safety. Because undergraduate students may have limited clinical exposure to severe burns, targeted educational experiences are needed before they enter professional practice. Although structured initiatives such as the Emergency Management of Severe Burns course support training in initial burn care [7], this course is not routinely delivered face-to-face in Portuguese in Brazil. The corresponding national initiative, the Curso Nacional de Normatização de Atendimento ao Queimado, is offered by the Brazilian Burn Society as a stand-alone course rather than as a component routinely integrated into undergraduate nursing curricula.

Improving education on burn management is therefore a priority in health profession education. Simulation-based education provides a safe environment in which learners can develop clinical reasoning, decision-making, communication, and teamwork in complex clinical situations [10,11,12]. In burn care education, several simulation modalities—including technologically advanced manikins, simulated patients, and virtual reality—have demonstrated benefits such as greater accuracy in fluid resuscitation calculations and more rapid recognition of inhalation injury, as well as reduced anxiety, improved communication in critical situations, and stronger leadership and teamwork skills [11,13,14].

The adoption of advanced simulation technologies may be constrained by costs, infrastructure, and limited institutional access [7,11]. Scenarios combining simulated patients with realistic moulage may provide clinical realism and learner engagement without relying on computerised manikins or other technology-intensive resources [15]. Furthermore, clearly defined objectives, opportunities for problem-solving, feedback, and structured debriefing have been associated with more favourable educational experiences [16,17,18].

The literature on simulation-based burn care education consists predominantly of single-centre studies and frequently focuses on mass-casualty incidents or highly specialised procedures [7,13,16,19]. Few studies have described scenarios that were systematically developed, subjected to content validation, and replicated across different institutions. Educational strategies are therefore needed that combine a sound clinical basis, prudent use of resources, and the capacity for implementation across different educational settings.

To address this gap, we developed and established the content validity of a resource-conscious simulation scenario for the initial management of adults with burn injuries. Its contribution lies in combining systematic content validation with standardised implementation across institutions using readily available resources. This study aimed to evaluate its implementation across three Brazilian public universities by examining perceived simulation design quality, perceived educational effectiveness, student satisfaction and self-confidence, and the associations between simulation design and these educational outcomes.

## 2. Materials and Methods

### 2.1. Study Design and Setting

This multicentre study was conducted at Santa Catarina State University (UDESC), the Federal University of Mato Grosso (UFMT), and the Federal University of Mato Grosso do Sul (UFMS), located in three Brazilian states across the South and Central-West regions. It comprised two complementary phases. The first was a methodological phase involving the development of a clinical simulation scenario focused on the initial management of an adult with burn injuries, along with content validation in accordance with procedures recommended in the literature [20]. The second consisted of a multicentre, cross-sectional, post-intervention evaluation of the validated scenario involving undergraduate nursing students. This phase was reported in accordance with the Strengthening the Reporting of Observational Studies in Epidemiology (STROBE) statement.

The study commenced on 20 May 2024 with the content-validation phase, which was conducted over a 30-day period. The validated scenario was subsequently implemented, and data were collected from undergraduate nursing students at these institutions between May and June 2026. The planning and delivery of the simulation-based experience were guided by the International Nursing Association for Clinical Simulation and Learning (INACSL) Healthcare Simulation Standards of Best Practice [21].

### 2.2. Scenario Development and Content Validation

The scenario was developed for its target population, comprising nursing students in the advanced stages of their undergraduate education. It depicted the initial management, in an emergency care setting, of an adult with partial-thickness thermal burns affecting 32% TBSA, involving the anterior chest, neck, and left upper limb, together with signs suggestive of inhalation injury. Its content was informed by key recommendations concerning initial assessment, airway management, estimation of the total body surface area burned, fluid resuscitation, analgesia, prevention of hypothermia, and criteria for transfer to a specialist burn centre [22,23,24,25].

The scenario was structured using a previously validated template [26] and included preparation, briefing, overview, clinical case, scenario design and progression, expected actions, debriefing, and evaluation. Learning objectives were formulated according to Bloom’s taxonomy and the specific, measurable, achievable, relevant, and time-bound (SMART) criteria. The expected critical actions followed the sequence of initial burn care. They included halting the burning process; removing clothing and jewellery when indicated; assessing burn extent and recognising possible inhalation injury and clinical severity; administering high-concentration oxygen; monitoring the patient; obtaining large-bore intravenous access, preferably in an unburned area; initiating fluid resuscitation with crystalloid solution; providing analgesia; inserting a urinary catheter to monitor urine output; preventing hypothermia; and arranging transfer to a specialist burn centre. The complete expected-actions checklist is provided in Supplementary Material S1. These were expected actions within the scenario and were not assessed as objective measures of procedural or clinical performance.

Content validation was defined as the systematic expert assessment of whether the scenario components were relevant and representative of the intended learning objectives, target learners, and clinical context; it did not assess learners’ clinical competence. Because the scenario targeted nursing students and initial nursing care in a non-specialist emergency setting, content validity was assessed by nurses with experience in simulation-based education (SBE) or emergency nursing. Potential experts were identified through the researchers’ professional networks and snowball sampling. Expertise was verified using a predefined scoring system adapted to the fields of clinical simulation and emergency care [27]. The system captured complementary dimensions of academic qualifications, research activity, clinical experience, and simulation-based education. Four points were awarded for a master’s degree in Nursing; two points for a doctoral degree in Nursing with a thesis related to either field of interest; one point for a master’s dissertation related to these fields; two points for conducting research; two points for publishing a scientific article; two points for a specialist qualification in emergency care or training as a clinical simulation instructor; and one point for at least one year of experience in emergency clinical practice or in the development, facilitation, or evaluation of clinical simulation activities. To join the expert panel, individuals were required to achieve a minimum of 5 points and demonstrate at least 1 year of experience in SBE.

Expert recruitment and content validation began on 20 May 2024 and were completed within the subsequent 30 days. Twelve professionals returned the completed instrument. Before the validity indices were calculated, their eligibility was verified using the predefined scoring system. Two assessments were excluded because the experts did not achieve the minimum required score, and one was excluded because the professional experience criterion was not met, resulting in nine assessments being included. The experts evaluated seven sections of the scenario using a four-point scale ranging from 1 (‘not relevant or representative’) to 4 (‘relevant and representative’).

For each of the seven sections, the content validity index was calculated by dividing the number of experts assigning a score of 3 or 4 by the total number of experts. Values of 0.78 or higher were considered acceptable [20,28]. The overall scale-level content validity index based on the average method (S-CVI/Ave) was calculated as the mean of the indices obtained for the seven sections. Recommendations provided in the open-ended response fields were reviewed by the research team for clinical relevance, clarity, and educational appropriateness. Modifications considered pertinent were incorporated into the final version. Validation was completed in a single round because all sections met the prespecified criterion. The final version of the scenario is available as Supplementary Material S1.

### 2.3. Participants

The population of interest comprised undergraduate nursing students in the advanced stages of their education. Students were eligible if they were enrolled between the eighth and tenth semesters of an undergraduate nursing programme at one of the three participating universities and attended both the preparatory educational activity and the scheduled simulation session. Students who did not agree to participate or did not provide written informed consent were excluded from data collection. The accessible population comprised all 48 students who met these criteria; all agreed to participate and constituted the final sample. Of these, 16 were from Southern Brazil and 32 from Central-Western Brazil.

No a priori sample size calculation was performed because the sample comprised all eligible students who attended the scheduled activities and agreed to participate. Accordingly, inferential analyses were considered exploratory, focusing on estimating the magnitude and precision of the associations rather than drawing confirmatory conclusions based solely on statistical significance.

### 2.4. Simulation-Based Intervention

Students received preparatory materials based on the Brazilian Ministry of Health guidance for the emergency treatment of burns and attended an approximately 50 min interactive lecture. The materials addressed burn classification and total body surface area estimation, systematic initial assessment, recognition of inhalation injury and severity criteria, and initial interventions, including oxygen therapy, intravenous access, fluid resuscitation, analgesia, prevention of hypothermia, wound care, and referral to a specialist burn centre. Although local facilitators with subject-matter expertise delivered the lecture, all sites used the same teaching plan and presentation, which had been discussed and agreed upon in advance by the research team. The simulation was conducted within one week of the theoretical session.

Six simulation sessions were conducted: two in the South region and four in the Central-West region. Each session involved up to 12 students. Three volunteers participated directly in patient care in each scenario, for a total of 18 active participants, while the remaining 30 students served as observers. Observers used the scenario’s expected-actions checklist to guide their observations. Active participants and observers took part in the debriefing together and completed the study instruments individually after the activity.

The scenario was intentionally designed as a uniprofessional learning experience for nursing students. The physician was scripted to enter the simulated encounter only after the initial phase, allowing students to recognise possible inhalation injury and initiate time-critical interventions within their professional scope and local protocols, including oxygen administration, removal of jewellery and burned clothing, patient monitoring, intravenous access, and fluid resuscitation. Participants could contact the physician by telephone at any point if they considered medical advice or support necessary.

A simulated patient and moulage were used to simulate partial-thickness burns and signs suggestive of inhalation injury. The setting reproduced an emergency care unit equipped with a multiparameter monitor, oxygen supply, airway management equipment, an emergency trolley, intravenous catheters, crystalloid solutions, medication administration supplies, and urinary catheterisation equipment.

Neither computerised manikins nor proprietary technologies were used. Moulage materials, including make-up, simulated blood, and prepared clothing, were already available at the participating centres. The main additional expense was the employment of a simulated patient, estimated at BRL 180 (USD 35) per session. No formal cost analysis was undertaken.

Each institution had a local facilitator. The facilitators were members of the research team who had contributed to the development of the scenario and were distinct from the nine experts involved in content validation. All facilitators were nurses and university lecturers with doctoral degrees, experience in emergency care, and training in clinical simulation. Before the intervention, the facilitators attended an online meeting to standardise procedures and subsequently followed the same protocol for student preparation, environmental set-up, clinical progression, and debriefing. As the three institutions were in different states, different simulated patients were used at each site. All simulated patients followed the same script and received additional in-person training on the day of the activity.

The briefing lasted approximately 5 min, the scenario 15 min, and the debriefing 30 min. Debriefing was conducted according to the principles of Debriefing with Good Judgment [29].

### 2.5. Instruments and Outcomes

The primary outcome was perceived educational effectiveness, assessed using the overall score of the Brazilian version of the Simulation Effectiveness Tool—Modified (SET-M). Perceived simulation design quality, measured using the design characteristics subscale of the Brazilian version of the Simulation Design Scale (SDS), was the primary explanatory variable. Student satisfaction and self-confidence were defined as secondary outcomes, whereas the perceived importance of the design elements, measured with the SDS importance subscale, was treated as a secondary explanatory variable. No objective measures of knowledge, technical performance, or clinical competence were included.

The Brazilian version of the SET-M comprises 19 items across four domains: pre-briefing, learning, confidence, and debriefing. Response options were ‘do not agree’ (1), ‘somewhat agree’ (2), and ‘strongly agree’ (3), with higher scores indicating greater perceived educational effectiveness. Domain scores were calculated as the mean of the corresponding items. For the primary analysis, the mean score across all 19 items was also calculated and defined a priori as an overall composite index of perceived educational effectiveness. The Brazilian version demonstrated a four-domain structure and satisfactory internal consistency and was validated for both active participants and observers of simulation-based activities [30].

The Brazilian version of the SDS, known as the Escala do Design da Simulação, comprises 20 items organised into five domains: objectives and information, support, problem-solving, feedback and reflection, and fidelity. For each item, participants separately evaluated the characteristics of the simulation design and the importance attributed to the corresponding design element. Design characteristics were rated on a five-point scale ranging from ‘strongly disagree’ (1) to ‘strongly agree’ (5). The importance of each design element was rated on a five-point scale ranging from ‘not important’ (1) to ‘very important’ (5). Overall and domain scores were calculated as the mean of the corresponding items, with higher scores indicating a more favourable assessment of the simulation design or greater importance attributed to its components [31].

The Brazilian version of the Student Satisfaction and Self-Confidence in Learning Scale, known as the Escala de Satisfação de Estudantes e Autoconfiança com a Aprendizagem, comprises 13 items across two domains: satisfaction with current learning (5 items) and self-confidence in learning (8 items). Participants rated each item on a five-point scale ranging from ‘strongly disagree’ (1) to ‘strongly agree’ (5), with the midpoint representing a neutral response. Satisfaction and self-confidence scores were calculated separately as the mean of the corresponding items, with higher scores indicating greater satisfaction or self-confidence [32].

Immediately after the debriefing, participants also completed a demographic and educational questionnaire that addressed age, sex, institution, self-reported prior education or training in burn care, and prior experience with simulation. There were no missing data for any of the instruments.

### 2.6. Statistical Analysis

Analyses were performed using IBM SPSS Statistics, version 29.0. Categorical variables were summarised using absolute and relative frequencies, whereas quantitative variables were described using medians and interquartile ranges because of the asymmetry of their distributions, as assessed using the Shapiro–Wilk test in conjunction with visual inspection. The internal consistency of the overall scores was estimated using Cronbach’s alpha. Coefficients for domains comprising only two items were reported descriptively and interpreted with caution.

The primary association between the SDS design characteristics score and the overall SET-M score was examined using Spearman’s rank correlation coefficient, with 95% confidence intervals. Associations between the SDS design characteristics score and satisfaction and self-confidence, and between the SDS importance score and each of the three outcomes, were considered secondary analyses. The magnitude of the correlations was interpreted according to Cohen’s criteria [33]: coefficients ranging from 0.10 to 0.29 indicated a weak correlation or small effect; coefficients from 0.30 to 0.49 indicated a moderate correlation or medium effect; and coefficients of 0.50 or higher indicated a strong correlation or large effect.

Differences between the two Brazilian regions were explored using the Mann–Whitney U test. Effect sizes were estimated by converting the Z statistic obtained from the Mann–Whitney U test into Cohen’s d using the following formula: d = $\frac{2Z}{\sqrt{n-Z^{2}}}$. This procedure involved the direct conversion of the Z statistic into r [34], followed by the conversion of r into d. Effect sizes were classified according to Cohen’s criteria [33], with values from 0.20 to 0.49 considered small, values from 0.50 to 0.79 considered medium, and values of 0.80 or higher considered large.

To account for potential confounding factors, the association between the SDS design characteristics score and the overall SET-M score was examined using multiple linear regression. Variables with *p* < 0.20 in the bivariate analyses were entered into the multivariable model. To assess the practical relevance of the model’s explanatory power, the coefficient of determination (R^2^) was interpreted according to the thresholds proposed by Cohen [33], whereby values of 0.02, 0.13, and 0.26 represented small, medium, and large effect sizes, respectively.

Model fit was assessed by inspecting a plot of residuals against predicted values. The residuals were randomly distributed around the horizontal zero line, with no discernible pattern or trend, supporting the adequacy of the model fit. All tests were two-tailed, and the significance level was set at 5%. No data imputation was performed.

### 2.7. Ethical Considerations

The study was initially approved by the Research Ethics Committee of Santa Catarina State University on 8 February 2023 (approval no. 5,882,806/2023). A subsequent amendment, which broadened the overarching protocol from emergency nursing simulation scenarios to scenarios in other areas of nursing, was approved on 28 March 2025 (approval no. 7,472,146/2025). The study was also authorised by the other participating institutions. All experts and students provided written informed consent.

## 3. Results

### 3.1. Content Validation of the Scenario

The final expert panel comprised nine nurses. Their mean age was 41.3 years (SD = 7.6), the mean time since graduation was 17.9 years (SD = 6.5), and the mean length of experience in clinical simulation or emergency care was 8.8 years (SD = 6.3). All experts had experience in simulation-based education; five had experience in emergency care, eight had conducted research in these fields, and seven had published related scientific articles. The experts represented four regions of Brazil.

All seven sections of the scenario met the minimum criterion for content validity. The content validity indices were 0.89 for preparation, overview, and design/progression; 0.78 for briefing, clinical case, and debriefing; and 1.00 for evaluation. The S-CVI/Ave was 0.86. Based on the experts’ written recommendations, minor changes were made to the student roles, prerequisites, learning objectives, clinical information, actor instructions, environmental resources, scenario progression, cues, and expected actions. As all sections met the prespecified criterion, no further round of evaluation was conducted.

### 3.2. Participant Characteristics

The study included 48 nursing students: 16 (33.3%) from Santa Catarina State University, 6 (12.5%) from the Federal University of Mato Grosso, and 26 (54.2%) from the Federal University of Mato Grosso do Sul. The median age was 23 years (IQR: 21–25; range: 19–34), and 42 participants (87.5%) were female. Thirty-one students (64.6%) self-reported prior education or training in burn care, and 26 (54.2%) had prior experience with simulation (Table 1).

### 3.3. Simulation Design and Perceived Educational Outcomes

Participants rated the simulation design favourably, with a median SDS design characteristics score of 4.60 (IQR: 4.21–4.89). Feedback and reflection and fidelity had the highest median domain scores (5.00), followed by objectives and information (4.70; IQR: 4.05–5.00). Support had the lowest median domain score, although it remained high (4.50; IQR: 4.00–5.00). The importance attributed to the simulation design elements was also rated highly, with a median SDS importance score of 4.93 (IQR: 4.56–5.00).

The median overall SET-M score was 2.63 (IQR: 2.32–2.88) on a scale ranging from 1 to 3. The highest median scores were observed for the pre-briefing (3.00; IQR: 2.50–3.00) and debriefing (3.00; IQR: 2.80–3.00) domains, whereas learning had the lowest median score (2.33; IQR: 2.00–2.67). Students also reported high levels of satisfaction (4.80; IQR: 4.45–5.00) and self-confidence (4.38; IQR: 3.91–4.63).

Internal consistency was adequate for the overall SDS design characteristics score (α = 0.814), SDS importance score (α = 0.949), overall SET-M score (α = 0.897), satisfaction score (α = 0.859), and self-confidence score (α = 0.798). As expected, domains comprising fewer items had lower internal consistency coefficients (Table 2).

### 3.4. Primary and Secondary Analyses

Exploratory comparisons between regions identified statistically significant differences in the SDS design characteristics score (Z = 3.468; *p* < 0.001; Cohen’s d = 1.16), satisfaction (Z = 3.360; *p* < 0.001; Cohen’s d = 1.11), and self-confidence (Z = 2.814; *p* = 0.005; Cohen’s d = 0.89). All three comparisons indicated large regional effects favouring the Central-West region (Table 3).

Among the SDS design characteristics domains, the largest effect was observed for feedback and reflection (Cohen’s d = 1.46), whereas the smallest was observed for support (Cohen’s d = 0.41); the latter difference was not statistically significant (*p* = 0.163). For the SDS importance subscale, only the fidelity domain differed significantly between regions (Z = 3.371; *p* < 0.001; Cohen’s d = 1.11), with a large effect size. Among the SET-M domains, only learning differed significantly between regions (Z = 2.114; *p* = 0.034; Cohen’s d = 0.64), with a medium effect size. All domain-level differences favoured the Central-West region.

In the primary analysis, the SDS design characteristics score was strongly and positively correlated with the overall SET-M score (ρ = 0.557; 95% CI: 0.317–0.731; *p* < 0.001). The SDS design characteristics score was also strongly and positively correlated with satisfaction (ρ = 0.711; 95% CI: 0.529–0.831; *p* < 0.001) and self-confidence (ρ = 0.612; 95% CI: 0.390–0.767; *p* < 0.001). The SDS importance score was strongly correlated with the overall SET-M score (ρ = 0.610; 95% CI: 0.386–0.765; *p* < 0.001), moderately correlated with satisfaction (ρ = 0.432; 95% CI: 0.160–0.643; *p* = 0.002), and strongly correlated with self-confidence (ρ = 0.532; 95% CI: 0.284–0.713; *p* < 0.001) (Table 4).

**Figure 1:**
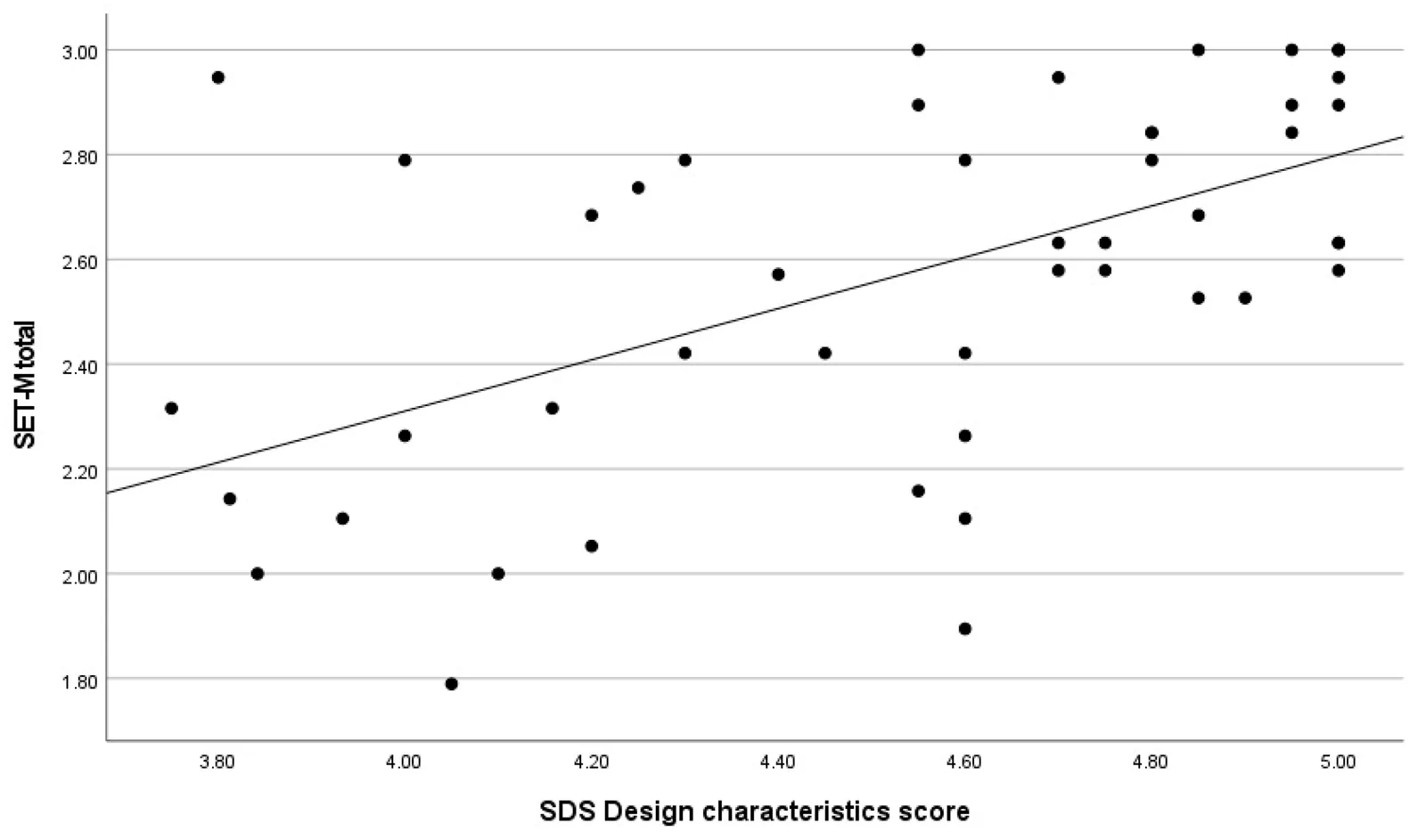
Association between the SDS design characteristics score and the overall SET-M score.

Figure 1 presents the scatter plot of the association between the SDS design characteristics score and the overall SET-M score.

In the exploratory analysis of associations between the SDS and SET-M domains, the strongest correlation was observed between the SDS problem-solving domain and the SET-M learning domain (ρ = 0.639; 95% CI: 0.427–0.785; *p* < 0.001), as shown in Table 5. The other statistically significant correlations were weak to moderate in magnitude, ranging from ρ = 0.299 (*p* = 0.044) to ρ = 0.495 (*p* < 0.001), with the strongest correlations involving the SET-M learning domain.

**Figure 2:**
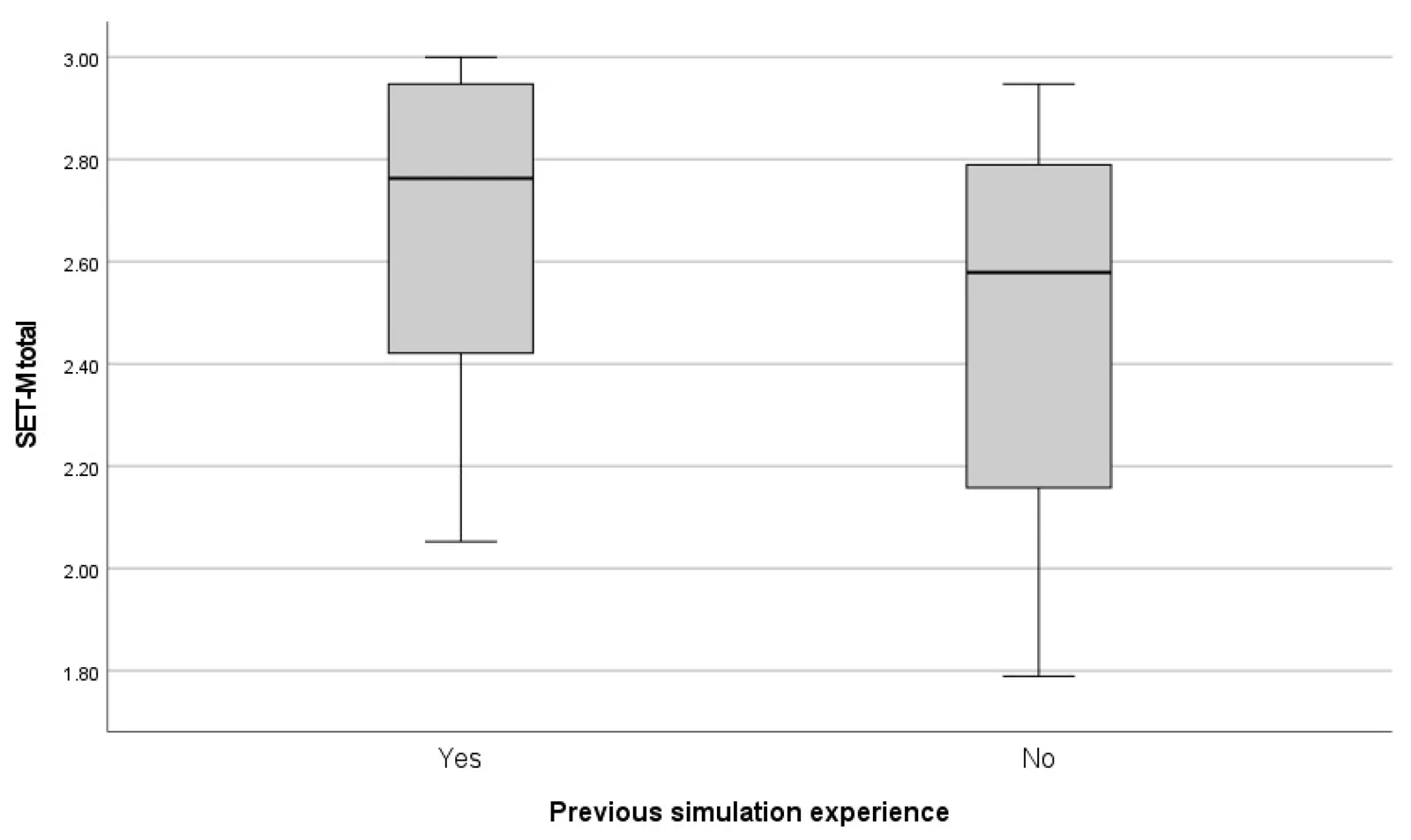
Overall perceived educational effectiveness (SET-M score) according to previous simulation experience.

The overall SET-M score was significantly higher among students with previous simulation experience (Z = 2.053; *p* = 0.040; converted Cohen’s d = 0.62), representing a medium effect size (Figure 2). Age was not significantly correlated with the overall SET-M score (ρ = 0.200; 95% CI: −0.034 to 0.513; *p* = 0.074).

Finally, a multiple linear regression model was fitted to examine the association between simulation design and perceived educational effectiveness. After adjustment for age, region, and previous simulation experience, each one-point increase in the SDS design characteristics score was associated with a 0.43-point increase in the overall SET-M score (b = 0.43; 95% CI: 0.17–0.69; *p* = 0.002). The model explained 31.3% of the variance in the overall SET-M score, corresponding to a large effect size after adjustment for the covariates included in the model.

## 4. Discussion

This study evaluated the multicentre implementation of a simulation scenario designed to teach undergraduate nursing students the initial management of adults with burns in settings with limited access to advanced simulation technologies. Students rated the simulation design, perceived educational effectiveness, satisfaction, and self-confidence favourably. The main finding was a strong positive association between perceived design quality and the overall SET-M score, together with similarly strong associations with satisfaction and self-confidence. The primary association remained after adjustment for age, regional grouping of the centres, and previous simulation experience, indicating that perceived design quality remained independently associated with perceived educational effectiveness. These findings suggest that pedagogical organisation is central to the educational experience, even when complex simulation technologies are unavailable.

This finding aligns with studies in different educational contexts reporting positive associations between simulation design characteristics, satisfaction, and self-confidence [17,35,36]. In the present study, the preparatory materials, standardised theoretical activity, briefing, observation guide, and structured debriefing may have contributed to the favourable ratings. These components can be adapted without advanced technological infrastructure.

The highest SDS scores were observed for Feedback/Reflection and Fidelity. The prominence of Feedback/Reflection is consistent with the central role of debriefing in simulation-based learning, as this phase enables learners to revisit decisions, articulate mental models, and relate the experience to clinical care [29]. The high SET-M debriefing score further indicates that students perceived this phase favourably. Although different debriefing approaches were not compared, the use of Debriefing with Good Judgment may have fostered a respectful environment of enquiry in which actions were discussed alongside the assumptions underpinning them. These findings are consistent with studies reporting high satisfaction when simulation provides structured feedback, faculty support, and opportunities for reflection [17,37].

The high Fidelity score despite the absence of computerised manikins suggests that fidelity resulted from the alignment of the simulated patient, moulage, emergency care environment, and structured clinical progression with the learning objectives. In burn simulations, moulage may enhance physical, conceptual, and emotional fidelity and learner engagement, although excessive stimuli can increase cognitive load [15]. This finding should nevertheless be interpreted cautiously because the SDS Fidelity domain comprises only two items and showed low internal consistency; therefore, its high median represents a favourable descriptive assessment rather than robust psychometric evidence [38]. Moreover, increasing technological complexity does not consistently improve all educational outcomes, as its effects vary according to the outcome assessed [39,40]. As the present study did not compare technologies or levels of fidelity, it cannot establish the superiority of this approach, but it shows that favourable perceptions can be achieved using a clinically grounded scenario and commonly available resources.

Within burn education, these findings extend a field characterised by heterogeneous objectives, populations, and modalities. A systematic review showed that simulation has been used in burn education, assessment, and management, but identified methodological diversity and limited outcome standardisation [7]. Models focusing on technical skills, burn assessment, and acute or surgical interventions predominated, whereas non-technical skills were addressed less frequently. The review also highlighted the need for validated, accessible models that can be integrated into educational programmes. In this context, the present study contributes a content-validated scenario focused on initial assessment and stabilisation that was tested across different institutions.

A randomised controlled trial found comparable outcomes for virtual reality and high-fidelity simulation in burn nursing care training, identifying virtual reality as a flexible and resource-efficient alternative [11]. A prospective study of a low-cost porcine model for burn excision and grafting also reported improvements in knowledge, confidence, and technical skills [41]. Although that study addressed surgical procedures and a different learner population, it further suggests that lower-cost solutions can offer educational value when aligned with the intended competencies. The scenario evaluated here prioritises recognition and initial stabilisation by generalist professionals, using a simulated patient and resources available in teaching facilities. Taken together, this evidence indicates that the simulation modality should be selected based on the learning outcomes, learner profile, and institutional resources rather than on technological complexity alone.

Favourable attitudes and perceptions towards simulation, including high satisfaction, self-confidence, and self-efficacy, have been reported across different educational contexts [36,37,42,43]. These outcomes reflect acceptance, engagement, and perceived ability to manage clinical situations; however, self-confidence does not directly equate to clinical competence, and SET-M scores represent perceived educational effectiveness rather than knowledge acquisition, retention, or objective performance.

The strongest domain-level association between SDS Problem Solving and SET-M Learning is pedagogically plausible. Initial burn management requires integrating airway and breathing assessment, estimation of burn extent, fluid resuscitation, analgesia, monitoring, and referral. Simulation allows learners to make these decisions sequentially and subsequently discuss them, thereby supporting clinical reasoning. A meta-analysis found that simulation-based interventions improve clinical decision-making among nursing students [10]. The progressive cues and expected actions may therefore have contributed to perceived learning by providing opportunities to analyse information and prioritise care.

Participation as an active team member or a guided observer was deliberate. Observers used a structured checklist, followed the expected actions, and participated in the debriefing, thereby maintaining cognitive and reflective engagement. Studies have shown that when observation is guided by objectives or structured instruments and followed by debriefing, observers may achieve outcomes similar to those of active participants in terms of learning, satisfaction, and self-confidence [44,45,46,47]. This arrangement also enables the strategy to be used with larger classes without requiring the scenario to be repeated for every small group.

Differences between centre groupings suggest that reproducing the same scenario does not eliminate the influence of local context. Facilitators, simulated patients, curricular experiences, class composition, and session dynamics may shape perceptions even when the script, theoretical presentation, and overall structure are standardised. As the Southern group comprised one university, whereas the Central-Western group included two universities and only six participants from one of them, the comparisons cannot be interpreted as an independent geographical effect and should remain exploratory. This heterogeneity reinforces that multicentre dissemination requires not only access to the scenario but also facilitator preparation, simulated patient training, and monitoring of implementation fidelity. Replicability does not imply identical scores, but rather the capacity to implement the essential components across different contexts.

Previous simulation experience was also associated with more favourable ratings of perceived educational effectiveness. Familiarity with the simulation environment, fidelity conventions, and debriefing may reduce the cognitive load associated with the format and allow greater focus on the clinical problem. As this analysis was exploratory, it is not possible to determine whether previous experience modified the response to the intervention or merely characterised students who were more familiar with and receptive to simulation.

The scenario can be integrated into undergraduate emergency nursing education and continuing or in-service training for professionals working in non-specialist emergency departments, particularly where access to advanced simulation technologies is limited. Using a simulated patient, moulage, and commonly available emergency equipment, it supports practice in recognising injury severity and possible inhalation injury, initiating oxygen therapy and fluid resuscitation, monitoring urine output, preventing hypothermia, and arranging transfer to a specialist burn centre. This approach may increase learners’ exposure to a severe but relatively infrequent emergency, but does not replace supervised clinical training or more complex technologies when required. Because clinical performance and patient outcomes were not assessed, its impact on the management of actual patients remains unknown and should be investigated in future studies.

### Strengths and Limitations

The study’s strengths include the systematic development and content validation of the scenario, its implementation across three public universities in different Brazilian states, and the standardisation of the intervention across centres. A common protocol, previously agreed preparatory materials and teaching plan, facilitator training, and guidance for simulated patients, helped preserve the essential components across settings. Further strengths include the use of instruments validated for the Brazilian context, the prespecification of the outcome and primary analysis, and the reporting of confidence intervals and effect sizes, allowing interpretation beyond statistical significance alone.

The findings should be interpreted in light of several limitations. The convenience sample was small and unevenly distributed across the three institutions. The expert panel comprised nurses with simulation and emergency-care expertise but did not include professionals specifically selected for specialist burn-care expertise, which may have limited the breadth of the clinical appraisal. The post-intervention design without a comparator was appropriate for this implementation stage and for evaluating perceived outcomes, but was not intended to estimate objective changes in knowledge or clinical performance; consequently, the favourable ratings should not be interpreted as evidence of clinical competence. Outcomes were self-reported and collected at the same time, making them susceptible to halo effects and common method variance. Although standardisation included a common protocol, facilitator training, and guidance for simulated patients, local differences in facilitation, performance, and session organisation could not be fully disentangled from institutional effects.

## 5. Conclusions

The content-validated scenario was implemented and evaluated across three Brazilian public universities, with favourable results for perceived design quality, perceived educational effectiveness, satisfaction, and self-confidence. More favourable perceptions of the design remained associated with better self-reported educational outcomes after adjustment for individual and contextual characteristics. The findings support the implementation of this clinically grounded, resource-conscious strategy across the three participating universities but do not establish clinical competence or superiority over other educational approaches. Future controlled studies using objective measures should evaluate knowledge, clinical performance, retention, and transfer of learning to practice.

## Figures and Tables

**Table 1:** Participant characteristics.

Variable	*n* = 48
Age, years, median (IQR)	23.0 (21.0–25.0)
Female sex, n (%)	42 (87.5)
Self-reported previous education or training in burn care, n (%)	31 (64.6)
Previous simulation experience, n (%)	26 (54.2)
**University, n (%)**	
Santa Catarina State University (UDESC)	16 (33.3)
Federal University of Mato Grosso (UFMT)	6 (12.5)
Federal University of Mato Grosso do Sul (UFMS)	26 (54.2)
**Region, n (%)**	
South	16 (33.3)
Central-West	32 (66.7)

**Table 2:** Perceived simulation design quality and perceived educational outcomes.

Instrument/Domain	Median (IQR)	Cronbach’s Alpha (α)
**SDS design characteristics score**	4.60 (4.21–4.89)	0.814
Objectives and information	4.70 (4.05–5.00)	0.735
Support	4.50 (4.00–5.00)	0.668
Problem-solving	4.60 (4.20–5.00)	0.625
Feedback and reflection	5.00 (4.75–5.00)	0.692
Fidelity	5.00 (4.50–5.00)	0.219
**SDS importance score**	4.93 (4.56–5.00)	0.949
Objectives and information	5.00 (4.45–5.00)	0.832
Support	5.00 (4.25–5.00)	0.848
Problem-solving	5.00 (4.45–5.00)	0.815
Feedback and reflection	5.00 (4.75–5.00)	0.932
Fidelity	5.00 (4.63–5.00)	0.895
**Overall SET-M score**	2.63 (2.32–2.88)	0.897
Pre-briefing	3.00 (2.50–3.00)	0.430
Learning	2.33 (2.00–2.67)	0.744
Confidence	2.67 (2.21–3.00)	0.878
Debriefing	3.00 (2.80–3.00)	0.642
**Satisfaction**	4.80 (4.45–5.00)	0.859
**Self-confidence**	4.38 (3.91–4.63)	0.798

Note: SDS, Simulation Design Scale; SET-M, Simulation Effectiveness Tool—Modified. Higher SET-M scores indicate more favourable perceptions (range: 1–3). Coefficients for domains comprising two items should be interpreted with caution.

**Table 3:** Perceived simulation design quality and perceived educational outcomes by university region.

Score	South Region, Median (IQR)	Central-West Region, Median (IQR)	Z	*p*	Converted Cohen’s d
**SDS design characteristics score**	4.28 (3.99–4.53)	4.78 (4.60–4.99)	3.468	<0.001	1.16
Objectives and information	4.40 (3.85–4.60)	5.00 (4.60–5.00)	2.926	0.003	0.93
Support	4.25 (4.00–4.83)	4.75 (4.00–5.00)	1.394	0.163	0.41
Problem-solving	4.20 (3.65–4.77)	4.80 (4.40–5.00)	3.382	<0.001	1.12
Feedback and reflection	4.50 (4.25–5.00)	5.00 (5.00–5.00)	4.077	<0.001	1.46
Fidelity	4.50 (4.00–4.88)	5.00 (5.00–5.00)	3.947	<0.001	1.39
**SDS importance score**	4.78 (4.14–4.99)	5.00 (4.61–5.00)	1.554	0.120	0.46
Objectives and information	4.80 (4.10–5.00)	5.00 (4.65–5.00)	1.434	0.152	0.42
Support	4.88 (4.06–5.00)	5.00 (4.25–5.00)	0.657	0.511	0.19
Problem-solving	4.70 (4.05–5.00)	5.00 (4.60–5.00)	1.517	0.129	0.45
Feedback and reflection	5.00 (4.13–5.00)	5.00 (5.00–5.00)	1.258	0.208	0.37
Fidelity	4.50 (4.00–5.00)	5.00 (5.00–5.00)	3.371	<0.001	1.11
**Overall SET-M score**	2.42 (2.19–2.78)	2.66 (2.53–2.93)	1.611	0.107	0.48
Pre-briefing	3.00 (2.50–3.00)	3.00 (2.50–3.00)	0.246	0.805	0.07
Learning	2.17 (1.88–2.46)	2.50 (2.21–2.79)	2.114	0.034	0.64
Confidence	2.50 (2.00–2.83)	2.75 (2.33–3.00)	1.673	0.094	0.50
Debriefing	3.00 (2.75–3.00)	3.00 (2.80–3.00)	−0.431	0.667	0.12
**Satisfaction**	4.30 (3.80–4.95)	5.00 (4.65–5.00)	3.360	<0.001	1.11
**Self-confidence**	4.06 (3.53–4.38)	4.38 (4.16–4.75)	2.814	0.005	0.89

Note: Mann–Whitney U test. SDS, Simulation Design Scale; SET-M, Simulation Effectiveness Tool—Modified; IQR, interquartile range.

**Table 4:** Associations between simulation design and perceived educational outcomes.

Explanatory Variable	Outcome	ρ	95% CI	*p*
SDS design characteristics score	Overall SET-M score	0.557	0.317–0.731	<0.001
SDS design characteristics score	Satisfaction	0.711	0.529–0.831	<0.001
SDS design characteristics score	Self-confidence	0.612	0.390–0.767	<0.001
SDS importance score	Overall SET-M score	0.610	0.386–0.765	<0.001
SDS importance score	Satisfaction	0.432	0.160–0.643	0.002
SDS importance score	Self-confidence	0.532	0.284–0.713	<0.001

Note: ρ, Spearman’s rank correlation coefficient; CI, confidence interval; SDS, Simulation Design Scale; SET-M, Simulation Effectiveness Tool—Modified.

**Table 5:** Associations between the Simulation Design Scale (SDS) domains and the Simulation Effectiveness Tool—Modified (SET-M) domains.

Explanatory Variable	Outcome	ρ	95% CI	*p*
SDS: Objectives and information	SET-M: Pre-briefing	0.352	0.067–0.584	0.014
SDS: Support	SET-M: Pre-briefing	0.387	0.107–0.610	0.007
SDS: Problem-solving	SET-M: Pre-briefing	0.179	−0.119–0.448	0.224
SDS: Feedback and reflection	SET-M: Pre-briefing	0.024	−0.274–0.317	0.875
SDS: Fidelity	SET-M: Pre-briefing	−0.135	−0.411–0.163	0.360
SDS: Objectives and information	SET-M: Learning	0.495	0.238–0.688	<0.001
SDS: Support	SET-M: Learning	0.478	0.216–0.675	<0.001
SDS: Problem-solving	SET-M: Learning	0.639	0.427–0.785	<0.001
SDS: Feedback and reflection	SET-M: Learning	0.418	0.140–0.635	0.003
SDS: Fidelity	SET-M: Learning	0.200	−0.097–0.465	0.172
SDS: Objectives and information	SET-M: Confidence	0.414	0.138–0.630	0.003
SDS: Support	SET-M: Confidence	0.361	0.077–0.591	0.012
SDS: Problem-solving	SET-M: Confidence	0.468	0.204–0.669	<0.001
SDS: Feedback and reflection	SET-M: Confidence	0.246	−0.053–0.504	0.096
SDS: Fidelity	SET-M: Confidence	0.065	−0.231–0.351	0.659
SDS: Objectives and information	SET-M: Debriefing	0.276	−0.024–0.531	0.063
SDS: Support	SET-M: Debriefing	0.460	0.187–0.667	0.001
SDS: Problem-solving	SET-M: Debriefing	0.299	0.000–0.548	0.044
SDS: Feedback and reflection	SET-M: Debriefing	0.084	−0.219–0.373	0.577
SDS: Fidelity	SET-M: Debriefing	−0.085	−0.374–0.218	0.572

Note: ρ, Spearman’s rank correlation coefficient; CI, confidence interval.

## Data Availability

The dataset supporting the findings of this study is not publicly available because it contains sociodemographic characteristics and institutional identifiers that may pose a risk of indirect participant identification, particularly given the relatively small sample sizes from each participating institution. A suitably de-identified dataset may be made available upon reasonable request to the corresponding author, Prof. Dr William Campo Meschial, subject to consultation with the Research Ethics Committee of Santa Catarina State University (CEP/UDESC) and compliance with applicable ethical and institutional requirements.

## Supplementary Materials

The following supporting information can be downloaded at: https://www.mdpi.com/article/10.3390/ebj7040052/s1, Supplementary Material S1: Resource-conscious clinical simulation scenario for the initial management of an adult with burn injuries.

## Abbreviations

The following abbreviations are used in this manuscript:

BRL	Brazilian real
CAAE	Certificate of Presentation for Ethical Appraisal
CEP/UDESC	Research Ethics Committee of Santa Catarina State University
CI	Confidence interval
INACSL	International Nursing Association for Clinical Simulation and Learning
IQR	Interquartile range
SBE	Simulation-based education
S-CVI/Ave	Scale-level content validity index based on the average method
SD	Standard deviation
SDS	Simulation Design Scale
SET-M	Simulation Effectiveness Tool—Modified
SMART	Specific, measurable, achievable, relevant, and time-bound
STROBE	Strengthening the Reporting of Observational Studies in Epidemiology
TBSA	Total body surface area
UDESC	Santa Catarina State University
UFFS	Federal University of Fronteira Sul
UFMS	Federal University of Mato Grosso do Sul
UFMT	Federal University of Mato Grosso
USD	United States dollar
