# Supplementary material for: Perceived Simulation Design Quality, Educational Effectiveness, Satisfaction, and Self-Confidence in a Resource-Conscious Burn Care Simulation: A Multicentre Observational Study
Source: Eur Burn J. 2026 Oct 9;7(4):52. doi: 10.3390/ebj7040052 (PMC13692479; doi:10.3390/ebj7040052)
Supplement: Supplementary file 1 [file ebj-07-00052-s001.zip › ebj-4543919-Supplementary Material S1.pdf]

Resource-Conscious Clinical Simulation Scenario for the Initial Management of an Adult with Burn Injuries

Institution: Santa Catarina State University – Nursing Department

Scenario developer: Prof. Dr. William Campo Meschial

Part 1: Prebriefing

Section I – Preparation

Preparation activities: A 50-minute interactive lecture on burn injuries and distribution of materials for advance study (Brazilian Ministry of Health burn care booklet).

Topics covered: Emergency management of a patient with burn injuries.

Additional activities on the day of the simulation: Presentation and discussion of an initial burn care algorithm.

Section II – Briefing

General guidance: Students observing the scenario should record questions and contributions for discussion during the debriefing. Participants are oriented to the simulated patient and to how assessments, vital-sign measurements, and procedures should be performed.

Duration: 15-minute scenario followed by a 30-45-minute debriefing.

Learner roles: Three students assume the role of nurses. The remaining students observe and assess the actions performed using a previously developed checklist.

Fiction contract: Participants are asked to engage actively and respond as they would in clinical practice. The activity provides a psychologically safe environment for decision-making and expression of opinions. Participants should acknowledge the constraints of the simulated environment while maintaining professional conduct and confidentiality.

Guidance for the simulated patient and accompanying relative: Respond only to questions asked by the learners and follow the actor script. If learners do not address a critical issue, scripted prompts may be used to provide a cue towards the expected actions.

Environment orientation: The scenario takes place in a fully equipped emergency room. Learners participating directly should be familiarised beforehand with the room, its equipment, and the emergency trolley.

Scenario aim: To provide the initial management of a patient with burn injuries while addressing the relevant technical and emotional aspects of care.

Part 2: Scenario Development

Section I – Overview

Scenario title: Initial Management of an Adult with Burn Injuries.

Target participants: Undergraduate nursing students.

Prerequisite: Completion of the first seven semesters of the undergraduate nursing programme.

Simulation modality: Clinical simulation involving a simulated patient.

Professional competencies: Clinical assessment; clinical reasoning; decision-making; communication; time management; and teamwork.

Overall learning objective: To develop the technical and scientific knowledge and critical-reflective reasoning required for the initial management of a patient with major burns and for effective teamwork.

Specific learning objectives: Perform a primary assessment using the ABCDE approach; recognise signs suggestive of inhalation injury; estimate the percentage of total body surface area (TBSA) burned; calculate the initial fluid requirement and commence intravenous crystalloid resuscitation; assess pain intensity and administer prescribed analgesia; assess the cutaneous burn injuries; prevent hypothermia; and arrange referral to a specialist burn centre.

Estimated duration: 15 minutes.

References:

Brazil. Ministry of Health. Emergency Treatment of Burns booklet. Brasília: Ministry of Health; 2012.

ISBI Practice Guidelines Committee. ISBI practice guidelines for burn care. Burns. 2016;42:953–1021.

ISBI Practice Guidelines Committee. ISBI practice guidelines for burn care, part 2. Burns. 2018;44:1617–1706.

American Burn Association. Advanced Burn Life Support Provider Manual. 5th ed. Chicago: American Burn Association; 2023.

American College of Surgeons Committee on Trauma. Advanced Trauma Life Support (ATLS): Student Course Manual. 11th ed. Chicago: American College of Surgeons; 2025.

Cartotto R, Johnson LS, Savetamal A, et al. American Burn Association clinical practice guidelines on burn shock

resuscitation. J Burn Care Res. 2024;45:565–589.

Section II – Clinical Scenario

Clinical case: A 27-year-old man sustained major thermal burns following a flash explosion while lighting a barbecue with liquid ethanol. He has deep partial-thickness burns involving the neck, anterior chest, and left upper limb, corresponding to approximately 32% TBSA, together with signs suggestive of inhalation injury. He presented immediately to an emergency care unit and is accompanied by his sister, who is visibly distressed and concerned. The learners assume the role of nurses in the emergency room.

Clinical guidance informing the scenario:

Brazil. Ministry of Health. Emergency Treatment of Burns booklet. Brasília: Ministry of Health; 2012.

ISBI Practice Guidelines Committee. ISBI practice guidelines for burn care. Burns. 2016;42:953–1021.

ISBI Practice Guidelines Committee. ISBI practice guidelines for burn care, part 2. Burns. 2018;44:1617–1706.

American Burn Association. Advanced Burn Life Support Provider Manual. 5th ed. Chicago: American Burn Association; 2023.

American College of Surgeons Committee on Trauma. Advanced Trauma Life Support (ATLS): Student Course Manual. 11th ed. Chicago: American College of Surgeons; 2025.

Cartotto R, Johnson LS, Savetamal A, et al. American Burn Association clinical practice guidelines on burn shock resuscitation. J Burn Care Res. 2024;45:565–589.

Section III – Scenario Design and Progression

Participants and roles: Three students assume the role of nurses.

Actors and roles: One simulated patient, one actor portraying the patient's sister, and one actor portraying the physician.

General instructions for the patient and relative: Do not volunteer information at the beginning of the scenario. Respond only to questions asked, in accordance with the script. The patient should groan and repeatedly report severe pain.

Actor 1 – simulated patient: At the start of the scenario, lie on the trolley, report severe pain, and ask the team for help. Simulate coughing and hoarseness. If asked about the accident, state that you were lighting a barbecue with liquid ethanol; when you poured the ethanol, the bottle caught fire and exploded. If asked about allergies, state that you have no known allergies. If asked about tetanus immunisation, state that you do not remember when you received your last dose. If asked about past medical history, state that you have no known health conditions.

Actor 2 – patient's sister: Remain beside the patient and appear visibly concerned. Ask the professionals to help your brother. If asked about the accident, state that you were inside the house when you heard him shouting and that he was already on fire when you reached him. If asked how the fire was extinguished, state that he rolled on the ground and you smothered the flames with a bath towel. When a learner introduces themselves as a nurse, ask where the physician is and how long it will take for the physician to arrive. When a learner reads Prompt Card 2, ask whether your brother will receive any medication. For questions not addressed above, respond, "I do not know" or "I did not see".

Actor 3 – physician: Remain outside the room until minute 8 of the scenario. If learners telephone before then, a nursing technician initially answers and explains that the physician is in the middle of a suturing procedure and will attend as soon as it is completed. If a learner explicitly asks to speak directly with the physician, take the call. Provide a verbal medication prescription only if the learner specifically requests medication or analgesia and communicates the relevant assessment findings. Enter the emergency room at minute 8, receive the learners' clinical handover, assess the threatened airway, and support the subsequent stabilisation and transfer plan.

Simulation environment: Emergency room in a 24-hour emergency care unit.

Equipment and Materials

Room/station resources: Trolley; table; fully stocked emergency trolley; multiparameter monitor; telephone; chairs; learners, facilitator, and actors; clipboard and pens; pain rating scale; airway and ventilation equipment; lactated Ringer's solution and intravenous administration supplies; simulated injectable medication vial; personal protective equipment; and infusion pump.

Moulage materials: Skin-safe liquid latex for theatrical make-up; thin tissue paper; cotton wool for blister formation; red, pink, brown, and black make-up pigments; simulated blood; sponges, brushes, and spatulas; black make-up to simulate soot around the nostrils; and pre-scorched clothing prepared for characterisation.

Moulage preparation: After screening for latex sensitivity, apply alternating layers of latex and thin paper over the left side of the chest and the left upper limb. Create irregular wound contours and areas resembling epidermal separation. Form blisters with small pieces of cotton wool covered with latex and paper. Once dry, apply pigments to reproduce erythema, variation in burn depth, and irregular margins, adding simulated blood where indicated. Complete the characterisation with pre-scorched clothing partially adhered to the moulage and black make-up around the nostrils to simulate soot.

Safety note: Clothing must be scorched away from the simulated patient and used only after it has completely cooled. Do not apply latex to individuals with a known latex allergy or to mucous membranes, damaged skin, or other contraindicated areas.

Resources for learners: Prompt Cards 1 and 2; conditional-deterioration information for the facilitator; fluid resuscitation

formula; adult Rule of Nines diagram; and performance checklist.

Resources for the simulated patient: Pre-scorched T-shirt; burn moulage; and make-up around the nose and mouth to simulate soot.

Prompt Cards, Starting State, and Cues

Prompt Card 1: The emergency physician is undertaking a procedure and is currently suturing another patient. The physician may be contacted on extension 4800. If learners call, a nursing technician initially answers and states that the physician is in the middle of the procedure and will attend as soon as it is completed. The call is transferred to the physician only if a learner explicitly asks to speak with the physician. The physician may provide a verbal medication prescription, but only when the learner specifically requests medication or analgesia. Provide this information at the start of the scenario.

Prompt Card 2: Heart rate 112 beats/min, regular rhythm; respiratory rate 19 breaths/min; SpO_2 96%; blood pressure 110/75 mmHg. Provide after the first set of vital signs is requested or measured.

Conditional deterioration: If learners fail to recognise the threatened airway, do not escalate care, or administer oxygen only through a nasal cannula, the patient develops visibly increased work of breathing, worsening cough and hoarseness, and greater distress. Provide the following observations when reassessed: heart rate 126 beats/min, regular rhythm; respiratory rate 25 breaths/min; SpO_2 92%; blood pressure 110/75 mmHg. If learners recognise the threat, administer high-flow 100% oxygen through a non-rebreather mask, and promptly contact the physician, the patient does not follow this deterioration pathway.

Physician arrival: The physician enters the emergency room at minute 8, regardless of whether telephone contact has occurred.

Starting state: The patient is positioned on a trolley in the emergency room, accompanied by his sister.

Facilitator cues: When requested by the learners, the facilitator provides information concerning the accident history, vital signs, and relevant clinical assessment findings. Scripted cues may be offered if learners fail to recognise a time-critical problem.

Performance Measures / Expected-Actions Checklist

No.	Expected action	Not performed	Inadequate	Partially adequate	Adequate
1	Welcomes and introduces themselves to the patient and accompanying relative.				
2	Commences multiparameter monitoring.				
3	Performs the ABCDE primary assessment and identifies immediately life-threatening problems.				
4	Requests the physician’s attendance or contacts the physician promptly.				
5	Assesses airway patency and looks for signs suggestive of smoke inhalation and impending airway compromise.				
6	Recognises suspected inhalation injury and administers high-flow 100% oxygen via a non-rebreather mask.				
7	Establishes two large-bore peripheral intravenous cannulae, preferably through unburned skin, and obtains blood samples as locally indicated.				
8	Commences warmed lactated Ringer’s solution at the calculated initial rate and plans titration to clinical response and urine output.				
9	Removes non-adherent burned clothing and jewellery and assesses the cutaneous injuries.				
10	Minimises heat loss and maintains a warm environment.				
11	Covers the patient with clean, dry sheets or blankets.				
12	Provides clear information and support to the accompanying relative.				
13	Estimates the burned area at approximately 32% TBSA using an appropriate method.				
14	Calculates the initial crystalloid requirement using 2 mL × 32 × 70 kg = 4480 mL, to be administered over the first 16 hours from the time of injury (initial rate: 280 mL/hour), with subsequent titration to urine output and clinical response.				
15	Inserts an indwelling urinary catheter when clinically indicated and monitors urine output.				
16	Administers prescribed intravenous morphine and reassesses pain and clinical response.				
17	Provides a structured clinical handover to the specialist burn centre and supports safe transfer arrangements.				

Section IV – Debriefing

Debriefing approach: Debriefing with Good Judgment.

Debriefing objective: To facilitate discussion and reflection on the competencies required of nurses during the initial management of a patient with burn injuries.

Suggested questions: Can you describe what happened? How did you feel during the scenario? Which actions went well? What would you do differently? What learning from this experience will you apply in future practice? Based on what you learnt today, what actions will you take? Summarise your main learning from today in one sentence.

Section V – Evaluation

Type of assessment: Formative.

Learner-performance instrument: Expected-actions checklist.

Participant evaluation instruments: Brazilian versions of the Simulation Design Scale, Student Satisfaction and Self-Confidence in Learning Scale, and Simulation Effectiveness Tool–Modified.
